# Novel Methacrylate-Based Multilayer Nanofilms with Incorporated FePt-Based Nanoparticles and the Anticancer Drug 5-Fluorouracil for Skin Cancer Treatment

**DOI:** 10.3390/pharmaceutics14040689

**Published:** 2022-03-22

**Authors:** Kristijan Skok, Tanja Zidarič, Kristjan Orthaber, Matevž Pristovnik, Nina Kostevšek, Kristina Žužek Rožman, Sašo Šturm, Lidija Gradišnik, Uroš Maver, Tina Maver

**Affiliations:** 1 Institute of Biomedical Sciences, Faculty of Medicine, University of Maribor, Taborska ulica 8, 2000 Maribor, Slovenia; kristijan.skok1@um.si (K.S.); tanja.zidaric@um.si (T.Z.); geminoduro@gmail.com (K.O.); mpristov147@gmail.com (M.P.); lidija.gradisnik@um.si (L.G.); 2 Department of Pathology, Hospital Graz II, Location West, Göstinger Straße 22, 8020 Graz, Austria; 3 Department for Nanostructured Materials, Jožef Stefan Institute, Jamova 39, 1000 Ljubljana, Slovenia; nina.kostevsek@ijs.si (N.K.); tina.zuzek@ijs.si (K.Ž.R.); saso.sturm@ijs.si (S.Š.); 4 Department of Pharmacology, Faculty of Medicine, University of Maribor, Taborska ulica 8, 2000 Maribor, Slovenia

**Keywords:** nanomaterials, bimodal therapy, topical skin treatment, magnetic nanoparticles, thin films, skin cancer, methacrylates

## Abstract

Despite medical advances, skin-associated disorders continue to pose a unique challenge to physicians worldwide. Skin cancer is one of the most common forms of cancer, with more than one million new cases reported each year. Currently, surgical excision is its primary treatment; however, this can be impractical or even contradictory in certain situations. An interesting potential alternative could lie in topical treatment solutions. The goal of our study was to develop novel multilayer nanofilms consisting of a combination of polyhydroxyethyl methacrylate (PHEMA), polyhydroxypropyl methacrylate (PHPMA), sodium deoxycholate (NaDOC) with incorporated superparamagnetic iron–platinum nanoparticles (FePt NPs), and the potent anticancer drug (5-fluorouracil), for theranostic skin cancer treatment. All multilayer systems were prepared by spin-coating and characterised by atomic force microscopy, infrared spectroscopy, and contact angle measurement. The magnetic properties of the incorporated FePt NPs were evaluated using magnetisation measurement, while their size was determined using transmission electron microscopy (TEM). Drug release performance was tested in vitro, and formulation safety was evaluated on human-skin-derived fibroblasts. Finally, the efficacy for skin cancer treatment was tested on our own basal-cell carcinoma cell line.

## 1. Introduction

The skin is the largest human organ, and as the first defence against external influences, it contributes to resisting pathogens and preventing water loss [1,2]. Therefore, any affliction to it has immense consequences, significantly affecting the quality of life. Despite medical advances, skin-associated maladies remain a unique challenge for physicians worldwide [3,4,5].

Skin cancer is a highly debilitating disease with major implications for global health. More than one million new cases are reported worldwide every year. The different types of skin cancer are named after the cells from which they develop and their clinical behaviour. The most common types are basal-cell carcinoma (BCC), squamous-cell carcinoma (SCC) (BCC and SCC are collectively referred to as non-melanocytic skin cancers (NMSCs), and present the most commonly diagnosed cancers), and malignant melanoma (MM) [6,7,8]. Skin cancer is commonly treated in six ways, i.e., by curettage and desiccation, surgical excision, radiotherapy, cryosurgery, topical therapy, and Mohs micrographic surgery, which is considered the gold standard. However, there are times when conventional therapies are impractical or contraindicated (e.g., cancer reoccurrence, removal may be harmful to the patient, metastases, etc.). Alternative treatment (e.g., chemotherapy) may be more efficacious in these cases, or might even present the only remaining treatment option [7,9]. The use of an active substance (e.g., chemotherapeutic) in combination with another therapeutic modality (e.g., magnetic hyperthermia) presents a promising approach in skin cancer treatment [10,11] which, despite recent advances, is still the most common malignant disease in Caucasians [6,12].

Recent advances in nanomedicine have revealed promise for creating new alternative treatment methods that are more efficient, minimise (severe) unwanted effects, and reduce treatment costs. Engineered nanomaterials are an attractive strategy for delivering therapeutic agents to the target tissue. These systems can be designed to overcome biological barriers in order to achieve maximum benefit with minimal unwanted effects (e.g., a lower dosage of therapeutic agents, increased efficacy, and fewer and/or less severe unwanted effects) [7,8,13]. Among a plethora of nanomaterials, nanoparticles (NPs) are among the most exploited nanocarriers in drug delivery systems. Apart from their protective function (i.e., protecting the encapsulated drug from degradation, absorption, metabolism, and excretion), they can improve distribution by limiting the distribution-to-blood volume. In addition, NPs offer the possibility of administering poorly water-soluble and hydrophobic drugs. The unique physical, chemical, and optical properties of NPs, as well as other nanomaterials, make it possible to image drug delivery in target tissues via non-invasive imaging and thermal or photo-controlled release [13,14].

Of special interest for our study are superparamagnetic nanoparticles (SMNPs), which offer magnetic treatment, retention, and manipulation in a controlled manner [15]. SMNPs in general include ferrous oxide particles (Fe_2_O_3_, Fe_3_O_4_), NiCu [16], FePd [17], and FePt [18] particles. Their properties enable multifunctional capabilities for imaging, drug delivery, and therapy. For example, coatings on ferrous oxide NPs can improve their stability, prevent agglomeration, and enable other functions (e.g., targeting, binding of active ingredients, etc.) [13,19,20]; they gain a large magnetic moment in an external magnetic field, and are considered superparamagnetic materials, making them interesting for biomedical applications [15,21]. Many of these properties are common and interchangeable between the aforementioned particles. As a result, SMNPs already meet the criteria for some clinical applications (e.g., image-guided nanocarriers for drug delivery). The development of different methods for surface functionalisation of SMNPs has enabled improved drug loading capacity and effective drug distribution (targeted delivery or controlled release). Moreover, magnetism not only makes them applicable as MRI contrast agents, but sometimes enables therapy with locally induced magnetic hyperthermia by converting external radiofrequency field energy into thermal energy [10,13,15,22,23].

Synthetic polymer dressings can be produced using various techniques, such as electrospinning, hydrogel synthesis, and spin-coating. Spin-coating is well known in the field of natural sciences; it has many favourable properties, which enable extensive modifications of the structure and properties of the product. This ensures the preparation of optimally defined products that can be easily evaluated using modern, highly specific techniques. Some more prominent examples of its application in the medical field include developing novel coatings for medical implants, medical devices, and various dressings [24,25,26]. Although multilayered and multifunctional topical dressings—especially those prepared via the layer-by-layer (LbL) technique—are not a novelty, existing products often do not address all of the challenging issues in topical delivery and treatment. Among the most challenging issues is achieving a controlled therapeutic effect while mitigating potential unwanted side effects [24,27]. We cannot overlook the potential of LbL in developing novel drug delivery systems that go beyond simple passive, diffusion-based delivery and offer different levels of control over the release and other functionalities [28,29,30]. A further step forward, and of particular interest to us, are new topical dressings incorporating NPs [17], which are promising as drug delivery systems for various forms of therapy, whether intravenous or topical [24,30,31,32]. Furthermore, NPs can add further modalities to the treatment and/or diagnostic options, and provide even more interesting treatment options.

Based on these considerations, we present here the development of multilayer nanofilms from a combination of polyhydroxyethyl methacrylate (PHEMA), polyhydroxypropyl methacrylate (PHPMA), sodium deoxycholate (NaDOC), fluorouracil (5-FU), and FePt NPs, using the spin-coating technique. 5-FU is an antimetabolite fluoropyrimidine analogue of the nucleoside pyrimidine with antineoplastic activity; it interferes with DNA synthesis by blocking the conversion of deoxyuridylic acid to thymidylic acid by the thymidylate synthetase. Topical 5-FU is often used for skin SCC when other treatments are not practical; it is particularly valuable in cases where postoperative healing is compromised. However, 5-FU’s highly hydrophilic nature limits its ability to penetrate the epithelium (through the *stratum corneum*) and reach the tumour tissue [7,9]. Considering the above, we predicted that this study’s specially designed multilayer dressing formulation would present an interesting and effective means of achieving prolonged exposure when administered topically. Specifically, the developed nanofilms seem very well suited for topical skin cancer treatment, where we would use the SMNPs for the incorporation and controlled release of 5-FU. At the same time, these NPs (with superparamagnetic properties) would enable us to track and evaluate this drug delivery system in the body through potential MRI imaging or other means to detect them after use. No multifunctional multilayer polymer/NP-based nanofilm dressing for topical skin cancer treatment has been previously reported, to the best of our knowledge. All prepared samples were characterised by atomic force microscopy (AFM), infrared spectroscopy (IR), and contact angle measurement. The magnetic properties of the incorporated FePt NPs were evaluated using magnetisation measurement, while their size was determined using transmission electron microscopy (TEM). Drug release performance was evaluated by an in vitro release test, and formulation safety was assessed on human-skin-derived fibroblasts. Finally, efficacy for skin cancer treatment was tested against a basal-cell carcinoma cell line isolated and established in our laboratories.

## 2. Materials and Methods

### 2.1. Materials

Polyhydroxyethyl methacrylate (PHEMA), polyhydroxypropyl methacrylate (PHPMA), sodium deoxycholate (NaDOC), sulfuric acid (H_2_SO_4_), and hydrogen peroxide (H_2_O_2_) were purchased from Sigma-Aldrich (Darmstadt, Germany), while absolute ethanol (EtOH) and 5-fluorouracil (5-FU) were purchased from a subsidiary of Sigma-Aldrich (Lyon, France). For the synthesis of the FePt NPs, platinum acetylacetonate Pt(acac)_2_ (Merck, NJ, USA), iron acetylacetonate Fe(acac)_3_ (>99.9%, Sigma-Aldrich, Lyon, France), benzyl ether (>98%, Merck, Branchburg, NJ, USA), oleic acid (OA; >99%, Sigma-Aldrich, Lyon, France), oleylamine (OLA; 70%, Sigma-Aldrich, Lyon, France), 1,2-hexadecanediol (90%, Sigma-Aldrich, Lyon, France), hexane (>95%, Sigma-Aldrich, Lyon, France), ethanol absolute anhydrous (>99.9%, Carlo Erba Reagents) (THF; anhydrous, >99.9%, Sigma-Aldrich, Lyon, France), hydrocaffeic acid (HCA; 3-(3,4-dihydroxyphenyl)propionic acid, >98%, Sigma-Aldrich, Lyon, France), and NaOH (anhydrous, >98%, Sigma-Aldrich, Lyon, France) were used. From Topsil (Winsen, Germany), atomic flat silicon wafers (Si wafers) were acquired, which were used as a substrate to prepare multifunctional thin films. All materials were used as supplied, without any further modification before sample preparation or testing. Ultrapure water (18.2 MΩ cm at 25 °C) was used to prepare water-based solutions, which were prepared with an ELGA PURELAB water purification system (ELGA LabWater, Veolia Water Technologies, High Wycombe, UK).

Human-derived skin fibroblasts (ATCC-CCL-110, Detroit 551) were obtained from LGC Standard (Bury, Lancashire, UK) and used for biocompatibility testing of the prepared samples. Human basal-cell carcinoma cells, which were used to evaluate the therapeutic efficacy of the proposed formulation, were isolated in our laboratory within the IRP-2014/01-35 project. Advanced Dulbecco’s Modified Eagle Medium (ADMEM), Advanced Dulbecco’s Modified Eagle Medium/Nutrient Mixture F-12 (ADMEM/F-12), and foetal bovine serum (FBS) were obtained from Thermo Fisher Scientific (Schwerte, Germany). Phosphate-buffered saline (PBS), bovine serum albumin (BSA), L-glutamine, penicillin G sodium salt, and streptomycin sulphate salt, as well as MTT tetrazolium salt ((3-(4,5-dimethylthiazol-2-yl)-2,5-diphenyltetrazolium bromide)), AlamarBlue (AB) reagent, CytoPainter Phalloidin-iFluor 555, and Fluoroshield with DAPI, were all purchased from Sigma-Aldrich (Darmstadt, Germany).

#### 2.1.1. Synthesis of FePt NPs

To synthesise FePt NPs, 0.5 mM Pt(acac)_2_ and 1 mM Fe(acac)_3_ were added to a round-bottomed flask containing 20 mL of benzyl ether at room temperature. Then, 4 mL of oleic acid and 4 mL of oleylamine were added. Before heating to 260 °C for 30 min, the reducing agent 1,2-hexadecanediol (2.3 mmol) was added at 160 °C. The black product was precipitated by adding absolute ethanol and separated by centrifugation (6000 rpm/10 min), and then redispersed in hexane. The hydrophilic NPs were obtained via the ligand exchange reaction. The as-prepared hydrophobic FePt NPs (20 mg) were dispersed in 1 mL of THF. The ligand solution was prepared by dissolving 50 mg of HCA in 5 mL of THF. The hydrophobic NPs were added dropwise to the solution of the ligand (hydrocaffeic acid), and the reaction mixture was then stirred at 50 °C for 3 h to complete the reaction. After cooling the reaction mixture to room temperature, 0.5 mL of 0.5 M NaOH was added to precipitate the NPs, collected by centrifugation, and redispersed in water.

#### 2.1.2. Preparation of Solutions

Four different solutions were prepared: The first was a “base polymer” formulation of PHEMA/PHPMA/NaDOC, consisting of 1 wt.% PHEMA, 0.1 wt.% PHPMA, and 1 wt.% NaDOC. A 10 mg/mL solution of PHEMA was prepared in EtOH, while both solutions of PHPMA and NaDOC were dissolved in ultrapure water—the former at a concentration of 1 mg/mL, the latter at a concentration of 10 mg/mL. To prepare the PHEMA/PHPMA/NaDOC formulation, all three solutions were mixed at a ratio of 1:0.1:1. The second solution was drug-containing PHEMA/PHPMA/NaDOC_5-FU, which was prepared in the same way as PHEMA/PHPMA/NaDOC, followed by the addition of a 6 mg/mL 5-FU solution (dissolved in water) to obtain the final formulation (ratio 1:0.1:1:1). The last two remaining formulations were nanocomposites; to prepare the third solution (PHEMA/PHPMA/NaDOC/FePt), a 1 mL aqueous suspension of FePt NPs (1 mg/mL) was added to the 2.5 mL solution of PHEMA/PHPMA/NaDOC (same concentration as in PHEMA/PHPMA/NaDOC), while for PHEMA/PHPMA/NaDOC/FePt_5-FU, the drug 5-FU (1 wt.% in the final solution) was subsequently added. All solutions were freshly prepared directly before use.

#### 2.1.3. Substrate Preparation

We used silicon wafers (Si wafers) as a substrate to develop multilayer thin films because of their atomically flat surface, which does not significantly affect the morphology of the materials in the spin-coating process. Before spin-coating, the Si wafers were cut into pieces of 8 × 8 mm^2^ using a diamond blade and soaked for 15 min at room temperature in a “piranha solution” (98 wt.% H_2_SO_4_ and 30 wt.% H_2_O_2_ in a ratio of 70:30 v/v). The as-prepared substrates were again rinsed with ultrapure water and blow-dried in a stream of dry nitrogen of high purity (99.999 wt.% Messer, Ruše, Slovenia).

#### 2.1.4. Multilayered thin Film Preparation

For creating multilayer systems, we used a spin-coater (POLOS, SPIN 150i, SPS GmbH, Ingolstadt, Germany) and the three types of formulations mentioned above (in Section 2.1.1.). The quantities of the components used are listed in Table 1.

The LbL coating preparation using spin-coating was carried out as follows: The base substrate (Si wafer) was placed on the spin-coater. A 50 μL drop of the as-prepared solutions/formulations was applied to the substrate. After this step, the spin-coating parameters were adjusted to best match the preparation of a “visually” smooth and reflective sample surface. The optimal conditions and parameter settings for the selected LbL coating process are shown in Table 2.

**Figure 1:**
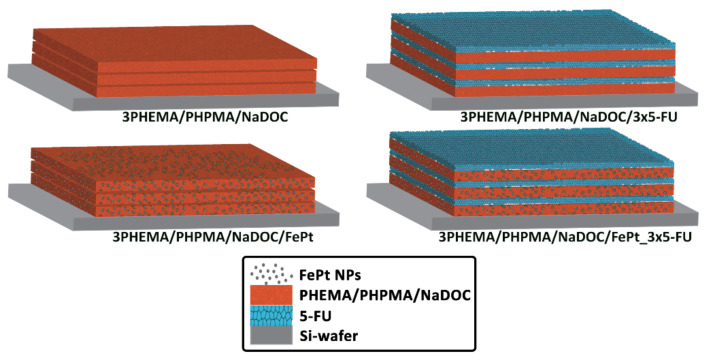
Schematic depiction of sample composition.

Under optimised settings, and following the same protocol, the process was repeated until multilayer coatings had formed on the Si wafer substrates. A similar procedure was used for the incorporated 5-FU: on the substrate coated with the first polymer layer (with or without FePt NPs), the second layer (and each subsequent uniform layer) was created by applying 50 μL of 5-FU solution. The preparation was completed by drying under high-purity nitrogen (99.999 wt.%, Messer, Ruše, Slovenia). The differently prepared multilayer thin film samples are schematically depicted in Figure 1.

### 2.2. Characterisation

#### 2.2.1. Contact Angle Measurement (Hydrophilicity/Hydrophobicity)

The hydrophilicity of the samples was investigated via water contact angle measurement (CA), which was performed using an OCA15Pro system (DataPhysics, Filderstadt, Germany) at room temperature. The sample masses (*m*), which change during the water adsorption phase as a function of time (*t*), were monitored. The initial slope of the function *m*^2^ = *f* (*t*) is known as capillary velocity, which can be used to determine the contact angle between the solid (polymer sample) and water using a modified Washburn equation [33,34]. All measurements were performed on three independent samples (each representative formulation on Si wafers) from three different sample areas. For this purpose, we applied a drop volume of 3 μL (in three replicates) of each sample. An average SCA value with the standard error was calculated using the hardware manufacturer’s software (software version 20.2.0).

#### 2.2.2. Attenuated Total Reflectance Infrared Spectroscopy (ATR-IR)

The ATR-IR spectra of spin-coated samples were recorded using an Agilent Cary 630 FTIR spectrometer (Agilent, Santa Clara, CA, USA) with a diamond ATR module with a measuring range of 400–650 cm^−1^. The scans were performed at three different locations in eight repetitions on each sample surface after preparation on the respective individual layer for each sample [33,35]. The IR permeability of the samples was evaluated using the software MicroLab PC 4.0 (Agilent, Santa Clara, CA, USA), and plotted as absorption (*y*-axis) against the wavenumber (*x*-axis). A resulting plot was additionally processed, aided with the OriginPro 8.5 (OriginLab, Stoke Mandeville, Buckinghamshire, UK).

#### 2.2.3. Atomic Force Microscopy

The surface morphology and the roughness parameters of the prepared samples were characterised by atomic force microscopy (AFM) in tapping mode with a Keysight 7500 AFM multimode scanning probe microscope (Keysight Technologies, Santa Barbara, CA, USA). The images were taken after drying the samples in a stream of dry high-grade (99.999 wt.%) nitrogen gas. The images were scanned with silicon cantilevers (ATEC-NC-20, Nanosensors, Wetzlar, Germany) with a resonant frequency of 210–490 kHz and a force constant of 12–110 N m^−1^. All measurements were performed at room temperature. All samples’ images of 10 × 10 μm^2^ with a resolution of 2048 × 2048 pixels [35] were recorded. The software Gwyddion (Czech Metrology Institute, Prague, Czech Republic) was used to process all images and calculate the corresponding roughness parameters.

#### 2.2.4. Magnetisation Measurement and Relaxometry

The synthesised FePt NPs (with average diameters of 6 ± 1 nm) were characterised for their superparamagnetic properties using a MicroSense FCM 10 vibrating sample magnetometer (VSM) [15,36] operated at room temperature (25 °C/285 K).

#### 2.2.5. Transmission Electron Microscopy (TEM)

The NPs were characterised using a (scanning) transmission electron microscope (TEM JEOL JEM-2010F) operating at 200 kV. Samples for the TEM analysis were prepared by adding a drop of NP suspension to a lacy, carbon-coated TEM grid at room temperature (25 °C/285 K) [36,37,38].

#### 2.2.6. Magnetic Resonance Imaging (MRI)

These results were obtained in our previous publications. The method, in brief, was as follows: Relaxation-time measurements [15,36] on the water suspension of FePt NPs were performed using an NMR/MRI system consisting of a 2.35 T superconducting magnet (Oxford Instruments, Abingdon, UK) and an Apollo NMR spectrometer (Tecmag, Houston, TX, USA). The *T_1_* relaxation times were measured with an inversion-recovery sequence with 16 different inversion times, ranging from 100 µs to 10 s, while the *T_2_* relaxation times were measured with the Carr–Purcell–Meiboom–Gill (CPMG) sequence with multiple spin-echoes. The *T_1_* and *T_2_* relaxation times were calculated from the best fits between the measurements and the corresponding model for either *T_2_* relaxation (exponential dependence of the echo signal on the echo count) or *T_1_* relaxation (dependence of the inversion recovery signal on the inversion time). The calculations were performed using the OriginPro 8.5 (OriginLab, Stoke Mandeville, Buckinghamshire, UK).

### 2.3. Functional Testing

#### 2.3.1. In Vitro Drug Release Testing

In vitro drug release was performed using an Automated Transdermal Diffusion Cells Sampling System (Logan System 912-6, Somerset, NJ, USA) [25,35]. The drug (5-FU)-containing samples were slowly introduced into the respective Franz diffusion cells (with an effective area of 3.14 cm^2^). The initial concentration of 5-FU in both drug-loaded samples (3PHEMA/PHPMA/NaDOC_3_5-FU and 3PHEMA/PHPMA/NaDOC/FePt_3_5-FU) was ~18,000 mg/mL, as a 5-FU-loaded polymer layer contained 6000 mg/mL 5-FU (see Table 1). The receptor compartment was filled with a phosphate buffer solution (PBS, pH = 7.4), and the temperature of the release medium was maintained at 37 °C. Stirring was constant at 50 rpm [25,35,39] throughout the entire in vitro drug release assay, which included sampling over 24 h at preselected time intervals (1, 5, 10, 20, 30, 60, 120, 180, 240, 300, 360, and 1440 min). The released/dissolved 5-FU concentration in the receptor medium was determined by UV–Vis spectrophotometry (Cary 60 UV–Visible Spectrophotometer, Agilent, Waldbronn, Germany), by quantifying the absorption band at 276 nm (characteristic of 5-FU) [40,41,42]. The removed sample volumes (of 1 mL) were replaced with fresh PBS, which was also tempered at 37 °C. The aqueous solubility of 5-FU [39,43] ensured that the released medium could be replaced with the same amount of fresh PBS buffer at sampling times to maintain sink conditions throughout the assay. These dilutions were taken into account when calculating the concentrations using the Beer–Lambert law [33,35]. Release studies were performed with at least three replicates, and results are reported as the mean with a standard error. To determine the total incorporated amount of 5-FU, all samples were placed in a glass beaker filled with 15 mL of PBS and sealed tightly. UV–Vis spectrophotometry was used to measure the amount of drug released daily until there was no change in concentration; this amount was used for further calculations.

To evaluate the release profiles, the in vitro drug release study results were fitted using a modified Korsmeyer–Peppas model that describes the release from polymer-based formulations [35,44].

#### 2.3.2. Cell Cultures and Viability Testing

A commonly used quantitative cell-based method was used to determine the potential cytotoxic effect (biocompatibility assay) of drug-free formulations. MTT assays based on the reduction of 3-(4,5-dimethylthiazol-2-yl)-2,5-diphenyltetrazolium bromide to formazan were used to evaluate the cell viability of human-derived skin fibroblasts. The applicability of such an assay in various samples is possible due to low background absorption in the absence of cells. There is a linear correlation between cell concentration and measured absorbance for each cell type, which is the basis for quantifying cell viability [33,45,46]. In this case, all drug-free samples (after sterilisation under UV light) were soaked in 3 mL of Advanced Dulbecco’s Modified Eagle Medium supplemented with 5 wt.% foetal bovine serum (ADMEM + 5 wt.% FBS), and incubated for 24 h at 37 °C in an atmosphere containing 5 wt.% CO_2_. Skin fibroblast cells (10,000 cells/well) were seeded in a 96-well (P96) microtiter plate with a final volume of 100 µL of ADMEM + 5 wt.% FBS. Supernatants of the initial samples and their dilutions (1:2, 1:4, 1:8, and 1:16) were added to the cells in four parallels after 24 h of incubation. The incubation was performed at 37 °C in an atmosphere containing 5 wt.% CO_2_. ADMEM + 5 wt.% FBS was added to the cells as a control. After 24 h of incubation, cell viability (i.e., cytotoxicity) was determined using the standard MTT assay. To assess the cell morphology of human skin fibroblasts growing on the thin films, phalloidin and DAPI staining were used to visualise the actin filaments and nuclei, respectively. The same experimental conditions were applied as for cell viability testing. After 24 h of incubation on the thin films, the medium was removed, and the cells were incubated with a fixative solution (Sigma-Aldrich) for 15 min at room temperature. After fixation, cells were washed with PBS (3 × 15 min) and then stained with CytoPainter Phalloidin-iFlour 555 (1:1000 in PBS containing 1% BSA) for 90 min at room temperature in complete darkness. Before imaging, the staining solution was removed, and the samples were covered with a drop of medium (Fluoroshield with DAPI) and incubated for 5 min in complete darkness. Imaging was performed using the EVOS cell imaging system.

**Figure 2:**
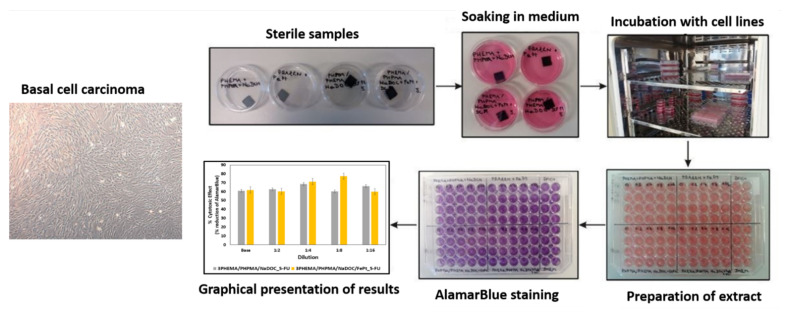
Isolated cell culture of human basal-cell carcinoma with schematics of the AlamarBlue assay.

To test the influence of the incorporated 5-FU on cell viability, it was studied on isolated human basal-cell carcinoma (BCC) cells with the chemical reduction of a resazurin-based reagent, which in oxidised form is a nontoxic, cell-permeable compound of blue colour without autofluorescence [47]. The AlamarBlue assay (Thermo Fisher Scientific Inc., Bremen, Germany), based on resazurin transformation, is designed to measure living cell proliferation and cytotoxicity. After entering living cells, resazurin is reduced to resorufin—a compound that is red and highly fluorescent. For use in this study, all samples were prepared to the size of 1 × 1 cm and sterilised under UV light for 30 min on each side. Samples were then soaked in 3 mL of Advanced Dulbecco’s Modified Eagle Medium supplemented with 5 wt.% foetal bovine serum (ADMEM/F12 + 5 wt.% FBS), and incubated for 24 h at 37 °C in an atmosphere containing 5 wt.% CO_2_. BCC cells (10,000 cells/well) were added to a 96-well microtiter plate (P96) with a final volume of 100 µL of ADMEM/F12 + 5 wt.% FBS, and incubated for 24 h at 37 °C in an atmosphere containing 5 wt.% CO_2._ Then, the same volume (100 µL) of the respective sample solutions and their dilutions (1:2, 1:4, 1:8, and 1:16) was added to the cells and incubated for 1 h at 37 °C in an atmosphere containing 5 wt.% CO_2_. All experiments were performed in four parallels. After 1 h of treatment, cell viability was determined by adding a 10 wt.% solution of AlamarBlue reagent (AB) at 37 °C in an atmosphere containing 5 wt.% CO_2_ to allow chemical reduction of the resazurin-based dye. Spectrophotometric detection of the resulting colour was performed using the Varioskan Multimode Microplate Reader (Thermo Fisher Scientific Inc., Bremen, Germany). At the predefined time points (2, 8, and 24 h), the samples were measured at a wavelength of 570 nm. At the second time point (24 h), additional spectrophotometric detection was performed at 600 nm. As controls, both ADMEM/F12 + 5 wt.% FBS and ADMEM/F12 + 5 wt.% FBS with 10 wt.% AB were added to the cells (in four parallels), and spectrophotometric measurements were performed after 24 h at both wavelengths (570 and 600 nm). The test was conducted according to the manufacturer’s protocol, where the absorbance for the control is part of the calculation to obtain the “percent difference in reduction”, according to [48]. Therefore, the control values were not drawn as part of the results. The results obtained are given as average values with corresponding standard errors. Due to the lack of the “usual” control, the statistical analysis was performed using single-factor analysis of variance (ANOVA), in which we compared the “percent differences in reduction” for the samples at 2 h (this was used as the “control” value) with the results after 8 and 24 h exposures. A *p*-value less than or equal to 0.05 was considered statistically significant. The isolated BCC cells and the schematic presentation of the viability assay are shown in Figure 2.

## 3. Results and Discussion

Cancer is a highly debilitating disease, and is expected to become even more important as life expectancy increases. Skin cancer is associated with the highest percentage of deaths, and despite numerous conventional therapies, these all have their respective limitations [7,14]. Therefore, improved skin cancer treatments are always needed. In this study, we created novel multilayer nanofilms as a platform for developing new theranostic (e.g., pharmacotherapeutic treatment and tracking of the drug delivery system) and/or multimodal (e.g., pharmacotherapeutic treatment combined with magnetic hyperthermia) skin cancer treatment strategies. To the best of our knowledge, this study represents the first demonstration of using spin-coated multifunctional nanofilms composed of the polymers mentioned above, a potent anticancer drug, and SMNPs in a theranostic skin cancer treatment.

### 3.1. Material Preparation

**Figure 3:**
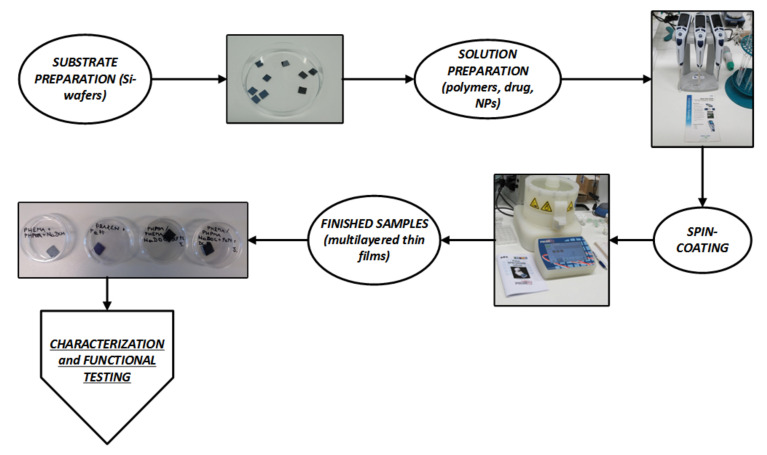
Schematic depiction of the step-by-step sample preparation procedure.

A major challenge in developing advanced therapeutic solutions is finding an effective delivery system that ensures the controlled release of bioactive components [49]. In this study, a polymer-based multilayer assembly (i.e., PHEMA, PHPMA, and NaDOC composite) was developed for this purpose. This combination was chosen based on our previous experiences with a similar composition but for a completely different purpose [26]. The multilayer nanofilms containing two active components—the superparamagnetic FePt NPs and the anticancer drug 5-FU—were prepared in LbL manner using spin-coating. The latter is a commonly used technique to prepare biomedically applicable coating materials, especially for multilayer systems; it offers several compelling advantages, including producing well-defined films, relatively easy state manipulation, and the production of materials with a wide range of properties. The methodology used herein was optimised based on our previous experiences related to multilayer film preparation [24,33,50]. Figure 3 depicts a schematic step-by-step preparation procedure for the prepared multilayer films.

The developed spin-coating process required several optimisation steps, especially considering the desired preparation repeatability. The first step was divided into two activities: (1) the optimisation of the spin-coating formulations (Table 2), and (2) the optimisation of the operating parameters (Table 3). The former was a prerequisite to successfully repeating the required number of respective samples without unnecessary intermission steps. At the same time, the optimisation of the process itself led to the formation of homogeneous films on the Si wafer substrate. The combined effect of both optimisations led to the fabrication of multilayer nanofilms with a smooth visual appearance for respective formulations. The final formulations were as follows: (1) *3PHEMA/PHPMA/NaDOC* (consisting of three polymer layers only), (2) *3PHEMA/PHPMA/NaDOC_3 × 5-FU* (consisting of three polymer layers and three 5-FU layers), as well as two samples containing the FePt NPs, (3) *3PHEMA/PHPMA/NaDOC/FePt*, and (4) *3PHEMA/PHPMA/NaDOC/FePt_3 × 5-FU*, the former without and the latter with three alternating layers containing the active substance (i.e., 5-FU).

### 3.2. Characterisation

#### 3.2.1. Hydrophilicity Evaluation through Contact Angle Measurement

Human skin is typified by a thick outer layer (i.e., *stratum corneum*) consisting of a complex “mixture” of proteins, lipids, and water-soluble molecules. For most substances, this outermost layer of the epidermis is regarded as the primary barrier against transdermal diffusion, and is also responsible for physicochemical features of the skin. The adsorption and absorption of pharmaceutical products into the human skin is influenced by numerous factors, including the wettability/hydrophilicity of the topical formulations [51]. The suitable hydrophilicity of materials used for topical (and transdermal) formulations is an important predictor of compatibility and adhesion to the skin and soft tissue substrates; furthermore, it is an important factor affecting drug release performance—especially from hydrophobic tablet matrices, films, and coated beads [52]. The contact angle measurement is usually regarded as the primary method for indicating the wetting behaviour of coatings and similar formulations. Contact angle measurements were performed in this study to evaluate the surface wettability of the prepared spin-coated multilayer nanofilms. As shown from the values in Table 3, all four formulations showed a hydrophilic character (values far lower than 90°), making them appropriate for human skin applications.

In the nanocomposite-based formulations (3PHEMA/PHPMA/NaDOC/FePt and 3PHEMA/PHPMA/NaDOC/FePt_5-FU), the somewhat lower contact angles (as opposed to the other samples with otherwise identical composition apart from the incorporated NPs) indicate improved wettability. The latter can be attributed to improved swelling properties due to strong interactions between incorporated FePt NPs and other components [53].

The initial moisture content can significantly influence the drug release kinetics due to porosity and changes in the base material’s mechanical strength and the *stratum corneum*’s physicochemical properties [51,54]. As 5-FU is hydrophilic and acidic [55], a decrease in contact angles was expected. Therefore, the obtained results are somewhat startling. When the 5-FU molecules were incorporated into the base material, the relative ratio of free hydroxy (-OH) groups, which can be used for inter- and intramolecular hydrogen bonding, changed in the base material (i.e., 3PHEMA/PHPMA/NaDOC and 3PHEMA/PHPMA/NaDOC/FePt), thus reducing the material’s ability to attract and bind water molecules [56,57]. With regard to the latter, the slight hydrophobicity of both 5-FU layers reduces the diffusivity of the incorporated drug molecules, thereby potentially allowing for a slower drug release.

Regardless of these considerations, the results confirm that the multilayer nanofilms produced, regardless of their composition, have suitable hydrophilicity to both control drug release and be used in skincare applications.

#### 3.2.2. Samples’ Structural Properties Evaluated by ATR-IR Spectroscopy

**Figure 4:**
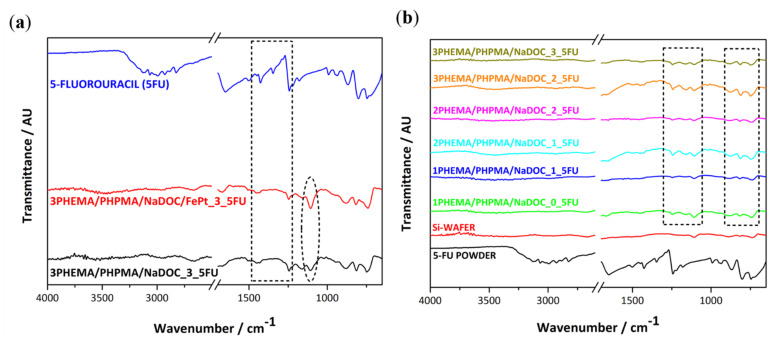
(**a**) IR spectra for the main sample components (5-FU, a sample composed of the polymer mixture with added 5-FU-3PHEMA/PHPMA/NaDOC_3_5-FU, and the sample with added FePt NPs-3PHEMA/PHPMA/NaDOC/FePt_5-FU). (**b**) IR spectra of our samples show the alternating manner, confirming the multilayer structure. The respective spectra are designated as xPHEMA/PHPMA/NaDOC_y_5-FU, where x and y represent the numbers of individual layers.

This study aimed to produce novel multilayer nanofilms with theranostic capabilities for skin cancer treatment. As shown below, these properties can be achieved by combining known biocompatible polymers, the potent anticancer drug 5-FU, and superparamagnetic FePt NPs. A multilayer structure was chosen for two reasons: Firstly, to ensure that the necessary dosage of the active components (5-FU and FePt NPs) to induce a pharmacotherapeutic effect can be deposited on the substrate in a patient-specific manner. Secondly, by adding the desired number of layers, the release behaviour of the dressings can be controlled. The multilayer nature of the prepared nanofilms was confirmed through the ATR-IR analysis after the preparation of the respective layers (Figure 4a). ATR-IR spectroscopy was performed for three main reasons: to detect the effective inclusion of (1) 5-FU molecules and (2) superparamagnetic FePt NPs, as well as (3) to confirm the successful preparation of the multilayer nanofilms (Figure 4b). Figure 4 also includes the spectra of pure 5-FU (using a drop of its ultrapure water solution) and the substrate (Si wafer), so as to better differentiate between the peaks in the individual layers, substrate, and active components.

From the recorded spectra shown in Figure 4, it can be seen that with the incorporation of the 5-FU layer, additional peaks between 1750 and 1500 cm^−1^ (marked with a dotted rectangle) appeared, which are most likely associated with the presence of amide I and amide III vibrations in the chemical structure of 5-FU [58,59]. For pure 5-FU, the absorption bands at 1725, 1672, and 1247 cm^−1^ belong to the aromatic ring (cyclic imide) and the vibration of imide stretching (amide I and amide III), respectively [60]. Since some of the intense characteristic peaks of the drug molecule do not occur at the same position in multilayer (nano)films, we can assume that strong chemical bonds between 5-FU and other compounds are present in the prepared samples [61,62]. The main reason for incorporating FePt NPs in spin-coating formulations was to enable controlled drug release and enable their detection and consistency during/after use (e.g., through MRI). Hence, IR spectra were measured to investigate the incorporated FePt NPs’ influence on the overall AFT-IR spectra and the method’s applicability for the other aforementioned purposes. Figure 4a,b also illustrate the corresponding IR spectra, in which all characteristic bands of PHEMA/PHPMA/NaDOC and 5-FU are shown. Under careful examination, a minimal change between the spectra after the addition of FePt NPs—specifically, below 1200 cm^−1^—can be seen (marked with the dotted ellipse). However, this change might not be enough to confirm the presence of the FePt NPs in the formulation. Nevertheless, based on the spectra, we can assume that the incorporated FePt NPs do not alter the chemical structure of the multilayer thin films, while the pharmacological properties of the 5-FU layer remain intact.

Figure 4b shows the IR spectra of the multilayer structure of 5-FU samples without FePt NPs (Figure 4a additionally shows that only one characteristic peak occurs through the incorporation of the NPs—marked with the dotted ellipse). The multilayer character of the respective films can be deduced from the appearance and/or disappearance of peaks (marked regions with the two dotted rectangles in Figure 4b) in alternating sequence—more pronounced after the second PHEMA/PHPMA/NaDOC layer (from blue spectra onward). In individual layers with a similar chemical composition, mainly typical peaks are visible. In contrast, the clearest and most distinct peaks could be assigned to the base material (i.e., PHEMA/PHPMA/NaDOC). These peaks correspond to stretching vibration of methylene (-CH_2_) groups around 2250 cm^−1^ and stretching bands for the ester bonds of the methacrylic and ethyl acrylic groups between 2100 and 1750 cm^−1^ [60,61].

Considering the alternative manner of occurrence of the respective peaks, which can be assigned either to the base material (i.e., PHEMA/PHPMA/NaDOC and PHEMA/PHPMA/NaDOC/FePt) or to the 5-FU, despite the application of additional layers (up to six layers), this confirms the proposed multilayer character of the prepared samples.

#### 3.2.3. Sample Surface Morphology and Roughness

**Figure 5:**
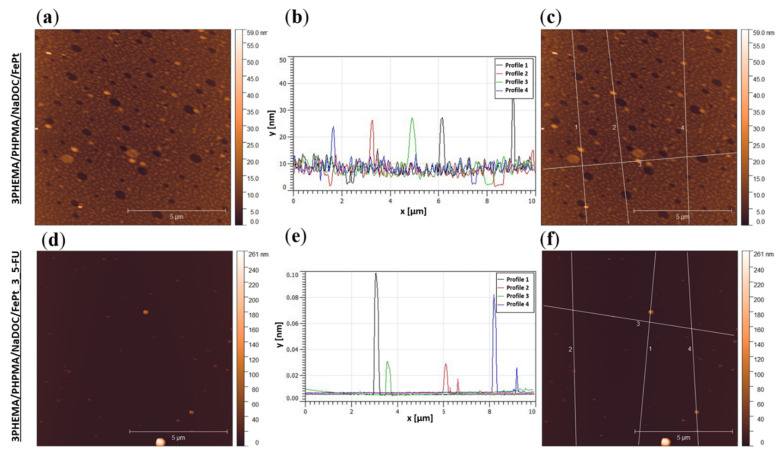
Images and surface profiles of samples recorded using AFM. The upper part of the image shows the results for the sample without the added drug: (**a**) topography, (**b**) surface profiles from which the size of the incorporated FePt particles can be determined, and (**c**) a picture indicating the locations where the profiles shown in (**b**) were recorded. The bottom part of the image shows the results for the sample with the added drug: (**d**) topography, (**e**) surface profiles from which the size of the built-in FePt particles can be determined, and (**f**) a picture indicating the location where the profiles shown in (**b**) were recorded.

Surface topography and roughness (on top of chemical composition) can influence the materials’ physicochemical properties, including hydrophilic behaviour, adhesive tension, and other properties, such as cell interactions [62,63]. AFM measurements were performed to analyse the surface morphology and evaluate the potential influence of incorporated active substances (i.e., 5-FU and FePt NPs) on the latter. The results are presented in Figure 5 as AFM images and profiles of the respective samples. For reasons of clarity and small-scale homogeneity, only measurements of the top layer for multilayer nanofilms (3PHEMA/PHPMA/NaDOC/FePt and 3PHEMA/PHPMA/NaDOC/FePt_3_5-FU) are presented in this subsection. At first glance at the obtained results, important differences in surface morphology between the two samples are visible, with a smoother morphology for the drug-free nanofilm (3PHEMA/PHPMA/NaDOC/FePt). These changes in surface morphology could be due to the differences in chemical characteristics and the preparation process itself. Specifically, the medium evaporates at different rates during the spin-coating process, affecting the sample’s morphology [25]. The differences in surface morphology shown in Figure 5 for the compared samples are also visible through the differences in surface roughness parameters between the two samples. Drug-free multilayer nanofilms show slightly reduced roughness parameters compared to those with 5-FU, most likely due to a different surface organisation of the respective layers (PHEMA/PHPMA/NaDOC/FePt or PHEMA/PHPMA/NaDOC/FePt_5-FU). In the PHEMA/PHPMA/NaDOC/FePt nanofilms, the polymer molecules were probably subject to supramolecular organisation in the form of spherical particles, visible as a relatively smooth layer on the surface. As opposed to the (probably) amorphous structure of the PHEMA/PHPMA/NaDOC/FePt layer, the 5-FU seems to “partially” form crystals. The mentioned crystalline form of the drug molecules, even if they are covered with another layer of PHEMA/PHPMA/NaDOC/FePt, influences the surface morphology of the sample considerably, which can be observed from the sharper edges of the surface features present on this sample. In this respect, the diluted 5-FU in 3PHEMA/PHPMA/NaDOC/FePt_3 × 5-FU recrystallized to some extent [33,64]. According to the literature, surface roughness increases the interface available for interactions, which is an important feature for the adhesion of the dosage form to the surface [62]. Thus, slightly increased roughness parameters in samples with 5-FU as the top layer favour the synergistic effect in sorption properties. Unfortunately, concrete roughness parameters, calculated based on the ISO 25,178 standard (commonly used to evaluate the whole surface roughness of samples [65]), could not be obtained for the prepared samples. Specifically, the visible particles (see Figure 5), which are a couple of times bigger than the expected height differences, distort these calculations, and do not give representative results for the roughness parameters. Overall, the AFM analysis showed that the proposed preparation method allows the fabrication of homogeneous surface films independent of the composition of the multilayer nanofilm.

Since AFM provides detailed information on morphological and structural changes of the surface (potentially even showing surface features resulting from structural changes in the bulk of the material), we also employed this technique to verify the presence of incorporated FePt NPs. Their presence in the formulation is crucial to ensuring that prepared multilayer nanofilms can provide multiple functions (e.g., FePt NPs with a superparamagnetic character enable potential tracking of their consistency after use). The latter is especially important considering that IR spectra were not conclusive enough to confirm the FePt NPs’ presence in the samples. The AFM images (Figure 5) show the presence of the FePt NPs in both samples. Each sample was measured in different areas to estimate the size of visible FePt NPs. As expected, the incorporated FePt NPs were in the size range of 10–50 nm [38], which is often considered the optimal size for nanoparticles in biomedical applications [11,15]. According to our results and the available related literature, the superparamagnetic FePt NPs most likely altered the topography of the prepared nanofilms by creating a magnetic field around them [64,66].

#### 3.2.4. Characterisation of the FePt NPs: Transmission Electron Microscopy (TEM), Magnetisation Measurement, and Relaxometry

**Figure 6:**
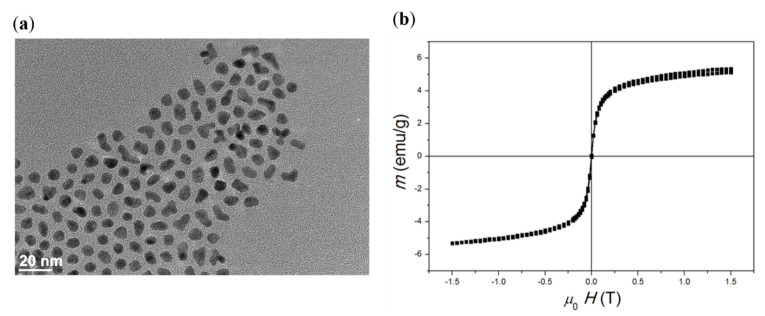
Characterisation of FePt NPs: (**a**) TEM image of as-synthesised FePt NPs. Particle size was determined to be 6 ± 1 nm (*n* = 40). (**b**) Magnetic measurement at 25 °C revealed the superparamagnetic shape of the curve and the saturation magnetisation of 12 emu/g.

The potential ability of SMNPs to act at the cellular and molecular levels due to their magnetic properties is known, and renders them interesting for targeted drug delivery and imaging [67,68]. However, to prove their suitability for the latter, two important properties are crucial, namely, the size and distribution and proper magnetic properties. To evaluate the former, the as-synthesised FePt NPs were studied using TEM. A representative TEM image of the FePt NPs (Figure 6a) shows well-dispersed NPs. The average size (defined as the average diameter) of FePt NPs calculated based on 40 individual particles was 6 ± 1 nm. This size range is appropriate for the particles to be used for tracking and imaging purposes but, unfortunately, too small to induce a therapeutic effect (i.e., magnetic hyperthermia) on their own—the required size to achieve such activity is above 15 nm [69].

To evaluate the magnetic properties of the prepared FePt NPs, and to test their response to a magnetic field, a vibrating sample magnetometer (VSM) was used. The saturation magnetisation of 6 nm FePt NPs was determined to be 12 emu/g (Figure 6b), which is consistent with previous results [15,36,70]. The absence of hysteresis is likely due to the fast Néel relaxation [67,71]. Since no hysteresis is generated (i.e., zero residual magnetisation remains after removing an external magnetic field), the probability of agglomeration in vivo is reduced [68]. The absence of remanent magnetisation suggests superparamagnetic properties of FePt NPs at room temperature. This makes them suitable in the prepared nanofilm dressing to achieve theranostic properties (together with the incorporated anticancer drug). Despite their being “too small” to achieve magnetic hyperthermia, these measurements nevertheless show their promise for use for this purpose in the future. Apart from the potential size increase (e.g., controlled agglomeration, prolonged nucleation), there are possibilities to achieve hyperthermia by other means (e.g., by coating them with SiO_2_ and Au, they could be heated up by using appropriate lasers as the stimuli) [15,72].

Research in local hyperthermia for cancer treatment mostly focuses on the use of active NPs, due to their ability to efficiently convert the external energy (e.g., laser light, magnetic field) into heat that is dissipated into the cellular environment [15,72,73]. Depending on the magnetic properties and surface effects, SMNPs can serve as MRI contrast agents, which means that cancer hyperthermia therapy and MRI can be performed simultaneously [74]. The MRI capability can be used to follow the potential release of NPs and/or to evaluate the extent of the drug release. Through this, the progress of the treatment can be monitored, improving its safety and efficiency [72]. In this context, the potential applicability of the herein-used FePt NPs as a MRI contrast agent was also investigated by measuring the dependence of longitudinal (*T_1_*) and transverse (*T_2_*) relaxation time on the concentration of FePt NPs (in water suspension) [36]. The values of both longitudinal (1/T_1_−1/T_1_(0)) and transverse (1/T_2_−1/T_2_(0)) relaxation rates increased for all samples, and based on the ratio of the calculated transverse (*r_2_*) and longitudinal (*r_1_*) relaxivity—i.e., the *r_2_*/*r_1_* ratio—a characterisation of the MRI contrast agent could be performed. The *r_2_*/*r_1_* ratio indicates whether a particular contrast agent can serve as a *T_1_* or *T_2_* contrast agent. Since SMNPs have high magnetisation values, they are classified as *T_2_* MRI contrast agents. A high *r_2_*/*r_1_* ratio (at least 10) is a prerequisite for an efficient *T_2_* MRI contrast agent [36,75,76]. In our case, the obtained *r_2_*/*r_1_* ratio was 52 [36], showing a promising role of FePt NPs in MRI imaging, consistent with previous studies [36,72]. In conclusion, the obtained measurements of magnetisation and relaxation indicate the potential of in situ incorporated FePt NPs in the prepared multilayer nanofilms to provide a vehicle for drug delivery and enable the evaluation of their consistency after application (e.g., through MRI).

### 3.3. Functional Testing

#### 3.3.1. In Vitro Drug Release Testing

The use of chemotherapeutic agents is one of the pillars of cancer therapy. Despite the progress and extensive research on new chemotherapeutic agents, their high cytotoxicity, which is not selective for cancer cells (i.e., it also affects normal cells), remains a dominant drawback. This cytotoxic effect is often severe, and it typically limits the dose administered; consequently, relatively low drug levels are often achieved in tumours [14]. By releasing the chemotherapeutic agent in a local, controlled manner, some of the unwanted side effects in the healthy surrounding tissue can be avoided [9]. Multilayer nanofilms are very promising in this regard, since they may effectively diminish drastic variations in concentration. This effect may even represent the difference between the desired therapeutic effect and local overdose, leading to significant damage to healthy tissue [33,77].

**Figure 7:**
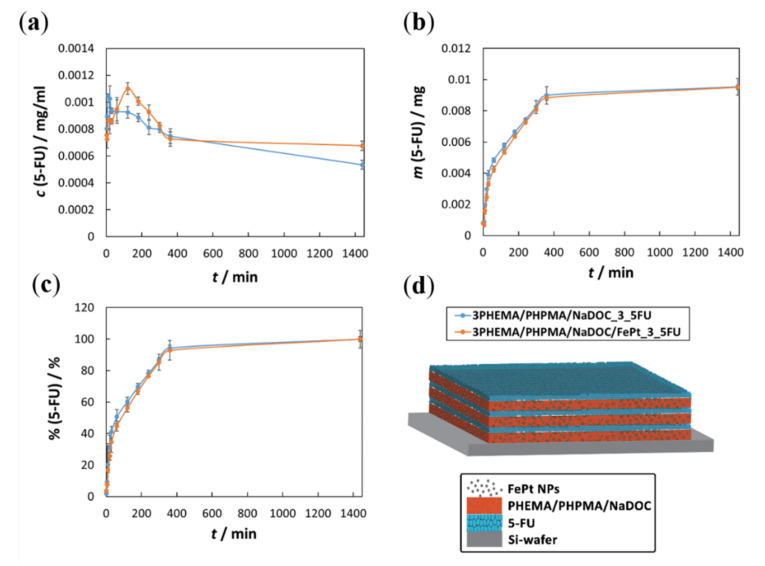
Results from the in vitro drug release testing: (**a**) released 5-FU concentration as a function of time: the fluctuating profile at the beginning of the release (at lower times) indicates a multi-layered structure. Each polymer layer acts as a barrier and somewhat retards the 5-FU release. As the latter disintegrates, the concentration rises again; (**b**) Cumulative released quantity of embedded 5-FU as a function of time: the apparent explanation of the cumulative released quantity is the presence of the incorporated FePt NPs in the second sample, which could be due to the interaction of the NPs with 5-FU, or the influence the particles might have on the incorporated 5-FU in the beginning. In both cases, the release slows down in the later times; (**c**) The percentage of released 5-FU as a function of time: the percentage between both samples is the same, which confirms the similarity in the releasing mechanisms, despite different compositions. It also confirms that the identical course of pharmacotherapy, despite different incorporated 5-FU dose; (**d**) schematic depiction of the multilayer structure of the prepared nanofilms.

In the scope of confirming the potential of the developed multilayer nanofilms for controlled drug release, in vitro drug release testing was performed. The relevant results are presented in the three respective graphs (illustrated in Figure 7), and explained below. For all curves shown, a confidence interval was determined as *± ts*/*√x*, where *t* is Student’s *t*-distribution, *s* is the standard deviation, and *x* is the number of measurements [25,35].

The changes in 5-FU concentrations as a function of release time during the in vitro release test are shown in Figure 7a. It can be seen immediately that a pulsatile release profile characterises the initial 360 min of release for both drug-loaded samples. Such a release profile reflects the multilayer structure of the sample [25,33,35,50], in which the drug and polymer layers (with or without FePt NPs) are deposited in an alternating manner (as schematically depicted in Figure 7d). Several peaks and valleys are observed in both samples, and the numbers of these peaks correspond to the numbers of drug-related layers in the respective sample. The release behaviour of 5-FU from both samples can be described as a “burst”-type release [25,33]. In this, 5-FU is released from the layer in direct contact with the medium. At the same time, underlying 5-FU layers contribute lower amounts of released 5-FU, resulting in an overall very fast release pattern in this part of the curve. After the initial time frame, a sustained drug release pattern is followed due to the underlying polymer layer acting as a barrier for the 5-FU and the next underlying layer. When the corresponding polymer layer is exposed to the medium, it may partially degrade or even peel off [25,33,35]. The process is repeated until all 5-FU layers are dissolved and the incorporated drug is depleted.

Considering these results, the drug release mechanism of 5-FU is most likely determined by the diffusion of drug molecules from the material into the release medium, which depends on the concentration gradient between the two mentioned areas. A smaller contribution to the overall release mechanism is due to the release of 5-FU from the underlying “5-FU layers” by slow diffusion through all layers above or lateral [25,33]. The acidic character of 5-FU also affects the release pattern. As mentioned above, the in vitro release studies were conducted at pH 7.4, at which 5-FU is ionised and negatively charged [78]. In addition, the enhanced interactions between 5-FU and functional groups of the polymer mixture (i.e., hydroxyl groups) are believed to be responsible for efficient drug encapsulation (and can further influence the release). All of the aforementioned, in turn, might be beneficial to ensure a low release during application [70,79]. The curves obtained in Figure 7a also indicate that incorporated FePt NPs have a negligible influence on the release mechanism in general, but might influence the cumulative dose of 5-FU in a given time. The latter can be attributed to a stronger interaction between the FePt NPs and 5-FU drug molecules compared to the polymer-only nanofilms. Specifically, in this case, closer bonds within the layer might be present and, through these (considering the negative charge of 5-FU at the pH of the release medium), increased repulsive forces [50,58].

Figure 7b shows the cumulative masses of the released 5-FU as a function of time. During the in vitro release tests, the withdrawn samples were replaced with fresh medium, and Figure 7b shows 5-FU masses as a function of time, taking into account these respective dilutions [25]. It is immediately apparent that the curves of both samples are almost identical, indicating that the release of 5-FU seems to be independent of the presence of FePt NPs. The relevance of this result lies in further confirmation that the proposed multilayer nanofilms can be used for theranostic skin cancer therapy (FePt NPs for targeting/tracking and 5-FU for pharmacotherapy). Another important (and related) conclusion is that both samples display an almost identical release profile, which is most pronounced in the initial part of both curves, where a “burst”-type release of 5-FU takes place after direct contact with the testing medium. This shows that a desired therapeutic drug concentration can be achieved rather quickly, leading to a potential rapid onset of activity. In general, the release mechanism can be divided into three different phases:

1. A fast (“burst”-type) initial release (the layer directly exposed to the medium for the first 30 min);
2. A sustained release, where the drug is released continuously (up to 360 min);
3. A slow release (i.e., release plateau), during which 5-FU is released from the remaining polymer layers that remained undissolved during dissolution (from 360 min to the endpoint of release).

These results indirectly confirm that the proposed preparation process allows for a simple approach to adjust the final dose of 5-FU. Regardless of the absolute amount of encapsulated 5-FU, all of the drug is released within a given time frame, achieving patient-specific drug dosing. From an economic point of view (i.e., high prices for numerous cancer drugs), such an approach could also benefit the healthcare system.

Finally, Figure 7c shows the obtained drug release results in the percentage of released 5-FU as a function of time. This percentage was calculated by dividing the amounts released after each time point by the final amount of 5-FU released. The endpoint was set after 1 day when the 5-FU concentration did not increase anymore [25,33]. The corresponding calculations complement the above results (Figure 7b). In particular, the release profiles for both samples follow almost identical release profiles (and, hence, mechanisms). Furthermore, from Figure 7c, it is evident that the inclusion of FePt NPs does not affect the release kinetics of 5-FU, indicating the suitability of such formulations with two incorporated “functional” components. As mentioned above, an important upgrade to the current FePt NPs lies in their functionalisation either to achieve bigger sizes and, hence, magnetic hyperthermia, or through coating them with Au in order to enable laser-induced hyperthermia. In such a case, the prepared nanofilms could be transformed from theranostic nanofilms to a bimodal therapeutic system.

Another important aspect to be considered in future applications is that the phase transition temperature of nanocomposites can be adjusted by modifying the monomers and their relative ratios in the polymer matrix. In this way, an accelerated dissociation of the polymer matrix can be achieved, facilitating the dissolution of 5-FU by increasing the water permeability, providing another means of potential release control. Due to the chemical potential gradient, this might contribute to the diffusion of the water-soluble 5-FU out of the polymer matrix [18,79,80]. The targeted response of the 5-FU to the tumour site and the improvement of its local uptake reduce unwanted side effects outside of the target area (tumour environment), and increase the therapeutic efficiency [81]. Considering the different cumulatively released 5-FU doses from each sample, this type of multilayer drug delivery nanosystem has the potential to ensure an identical course of therapy even with different doses. The latter is very important for patient-specific treatment, where the drug dose has to be adapted to the respective patient’s needs. From this, we can conclude that the prepared multilayer nanofilms have great potential for further studies as novel controlled drug delivery systems capable of achieving and maintaining patient-specific drug levels.

#### 3.3.2. Cell Cultures and Viability Testing

Cancer treatment commonly involves anticancer drugs known for their serious unwanted side effects. The search for safer treatment strategies remains a relentless challenge for researchers. For this reason, our study examined both the efficacy of the prepared samples against skin cancer cells and their safety through the exposure of human skin fibroblasts to the sample extracts, according to the ISO 10993-5 and -12 standards.

**Figure 8:**
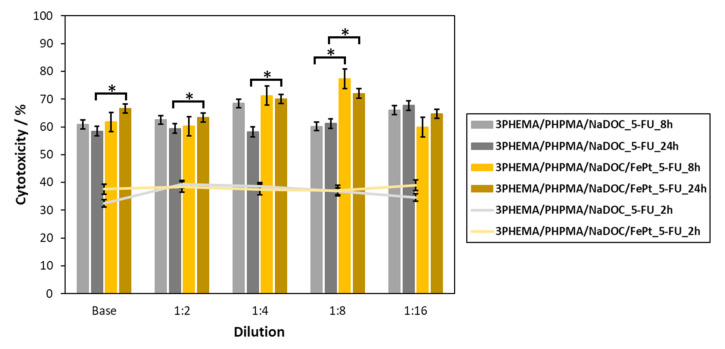
Cytotoxicity testing results on the basal-cell carcinoma cell culture. Grey-coloured columns show the results for samples with incorporated 5-FU only, while the yellow-coloured columns show the results for samples with incorporated 5-FU and the FePT NPs. The results show values calculated according to the manufacturer’s instructions for “percent difference in reduction”, which correspond to the cytotoxicity after 2, 8, and 24 h exposure of the BCC. Statistical significance (*p* < 0.05) was determined for all samples compared to the control (2 h BCC exposure; grey and yellow lines) (ANOVA test). Comparison of samples with (3PHEMA/PHPMA/NaDOC/FePt_5-FU) and without FePt NPs (3PHEMA/PHPMA/NaDOC_5-FU) was also evaluated, and * corresponds to statistically different samples in respective dilutions and exposure times (*p* < 0.05).

The efficacy of both developed drug-containing samples (3PHEMA/PHPMA/NaDOC_3_5FU and 3PHEMA/PHPMA/NaDOC/FePt_3_5FU) was evaluated on human basal-cell skin carcinoma cells (BCC) using the AlamarBlue^®^ assay. The goal was to show the anticancer activity as the reduction in cancer cell viability. During this testing, an additional evaluation point was to investigate the effects of the controlled release of 5-FU on the course of treatment. This is generally an important test parameter, since the latter can influence both therapeutic and adverse effects. To determine both effects, the cytotoxicity of all prepared samples was determined using the measurement of cell proliferation after two defined exposure periods (i.e., 8 h and 24 h) [82]. It is known that 5-FU has antitumour effects by inhibiting the nucleotide synthesis enzyme thymidylate synthase and causing the misincorporation of fluoronucleotides into nucleic acids. In this way, it can inhibit tumour cell growth by arresting malignant cells in the S-phase and forcing them to undergo apoptosis [83]. In addition, its cytotoxicity is strongly dependent on the exposure time, with a rising efficiency with prolonged exposure [84,85]. Considering this mechanism of action and the expected more pronounced effect on BCC viability at longer exposures, we chose to measure the effects of the samples after 8 and 24 h. Since the used protocol to calculate the “percent difference in reduction” according to the manufacturer’s instructions already includes the control sample’s values in the equation, and considering the effect of 5-FU requiring longer exposure times, we used another time point (2 h of exposure) for comparison with the results after 8 and 24 h of exposure, respectively. The results in Figure 8 are presented from the perspective of a dose-dependent (i.e., base concentrations and their dilutions) effect on cell viability. We can immediately observe that both samples significantly reduced (compared to the 2 h exposure) the cell viability of BCC by at least 60%, regardless of the “longer” exposure time.

Furthermore, it appears that incorporated FePt NPs (even without any external magnetic stimuli) enhance the anticancer properties of 5-FU multilayered nanofilms, which was also statistically significant after 8 h and 24 h of exposure (*p*-values of < 0.05), but for different dilutions. In the case of 8 h exposures, the differences were significant for the base sample and the 1:4 and 1:16 dilutions. In the case of 24 h exposures, significant differences were observed for all of the dilutions (and the base sample), apart from 1:16. One possible explanation might be in the acceleration sedimentation on the cell surface triggered by NPs, leading to a higher cellular uptake [86,87]. Despite the slight increase in cytotoxicity, this was mainly due to the 5-FU itself, and FePt NPs played only a limited role in the cells’ antitumour activity per se (e.g., apoptosis of BCC cells) [85]. The dominant antitumour effect of 5-FU is more evident in the long term. Comparing the average cytotoxicity percentages for both periods, as expected, an exposure of 24 h has a greater effect on cell viability—a trend that has also been observed previously [84,85]. This means that a reduced BCC viability is observed at all dilutions (all samples decreased the BCC viability). The reduced cell viability is most likely the consequence of the sustained release of 5-FU from the respective samples.

Surprisingly, regardless of the exposure time, there was a difference in the maximum cytotoxic effect for both samples. Specifically, in the case of the 5-FU- and FePt-NP-loaded samples, the highest cytotoxicity percentage against BCC was observed for the 1:8 dilution. On the other hand, in the case of the samples loaded with 5-FU only, the maximum cytotoxic effect was observed at the 1:4 dilution (for the 8 h exposure) and the 1:16 dilution (for the 24 h exposure). This phenomenon can be partly elucidated in terms of cellular activity and the release mechanism of 5-FU. Specifically, BCC has proliferative properties similar to those of other solid tumours commonly referred to as “fast diving” [88], and 5-FU is a purely S-phase drug with no activity when the cells are in the G0 or G1 phase [89]. Taking into account a “burst”-type release of 5-FU in the initial phase (as described in Section 3.3.1.), “concentrated” extracts (base concentration, dilutions 1:2 and 1:4) have an immediate cytotoxic effect on cells. In the case of a 1:8 dilution, the respective concentration was insufficient to yield superior cytotoxicity, so some fraction of the BCC in the S-phase might not be inhibited. Therefore, during the sustained part of the 5-FU release, more cells in the S-phase were more susceptible to 5-FU.

To evaluate the results in more detail, we also performed a statistical evaluation of the obtained results. Two types of calculations were performed, namely, the comparison of the short exposure times (2 h vs. 8/24 h), and the comparison of the samples with or without the added FePt NPs. This analysis revealed statistically significant (*p*-value < 0.05) changes in the cytotoxic effect after both longer lasting exposures (8 and 24 h) for all dilutions compared to the 2 h exposure. This result is consistent with the above consideration that the inhibitory effect of the 5-FU molecule in the polymer film becomes more evident with prolonged exposure [84,85]. Despite the somewhat contradictory results in the comparison between 5-FU samples with and without FePt, it is nevertheless clear that both sample types (regardless of dilution) have a pronounced cytotoxic effect. The analysis further revealed that the cytotoxicity against BCC is slightly better in the case of samples with the incorporated particles. This is true for most samples, as can be seen for several dilutions at both exposure times (Figure 8, marked with *). Considering this (and the discussion above), we can assume that FePt contributed to the overall performance of the films as a controlled “drug delivery system”. Although further studies might be needed in order to understand this phenomenon fully, the obtained results already seem promising. However, in order to fully validate the safety and antitumour efficacy of our proposed drug-loaded nanofilm samples, these encouraging results will also need to be supported by in vivo studies, which will be the next step in their evaluation.

**Figure 9:**
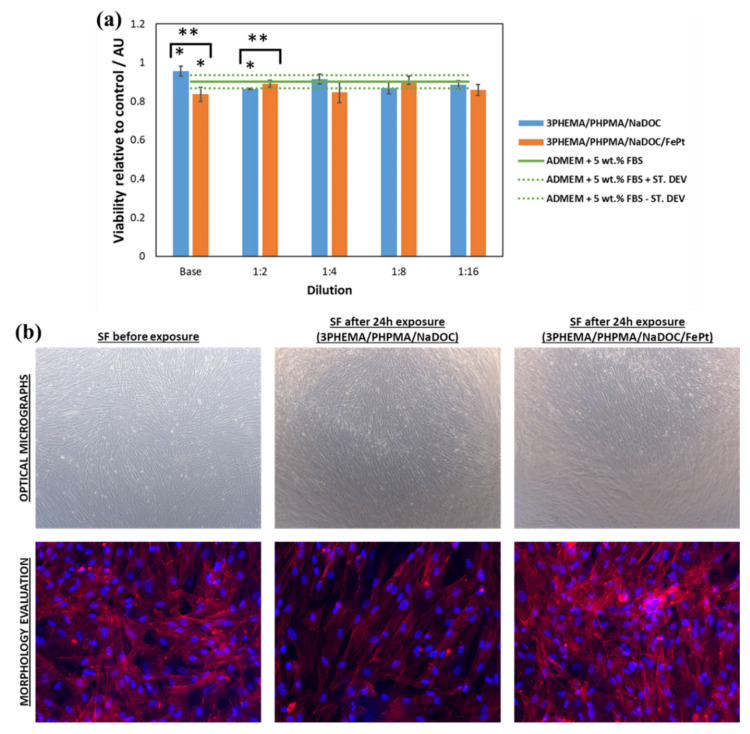
Human skin fibroblast biocompatibility test results: (**a**) metabolic activity in MTT assay of cells grown on drug-free nanofilms; and (**b**) optical micrographs and cell morphology after 24 h incubation of the cells in the sample extracts, which show no significant changes in cell morphology. Statistical significance was defined as * *p* < 0.05 for all samples compared to the control (ADMEM + 5 wt.% FBS—green line) (ANOVA test) and ** *p* < 0.05 for all 3PHEMA/PHPMA/NaDOC/FePt compared to the 3PHEMA/PHPMA/NaDOC.

Since the targeted application of our prepared multilayer nanofilms is in treating skin malformations, biocompatibility with healthy human skin fibroblasts was also investigated (Figure 9). In this case, the in vitro evaluation of cell viability was performed using the MTT assay. Both viability assays (the MTT and AlamarBlue assays) were used to test the intrinsic cytotoxicity of substances, but rely on slightly different mechanisms. The MTT assay measures cell viability by relying on intracellular NAD(P)H-dependent dehydrogenases, which convert the tetrazolium salt (MTT) to purple formazan crystals. The extent of the colour reflects the number of metabolically active cells present in a sample. In the case of the AlamarBlue assay, the reduction of its active ingredient (resazurin) to resorufin is thought to be mediated by mitochondrial enzymes, and the resulting colour intensity is considered proportional to the number of viable cells present in a sample [90,91]. Although both rely on intracellular metabolism as the means to evaluate viable cells, the MTT assay is an “acutely destructive” technique for the cells. Specifically, the formed formazan crystals destroy the cells in which they are formed. On the other hand, resorufin (formed in the AlamarBlue assay) does not cause any immediate critical damage to the cells); consequently, it can also be used for continuous experiments, in which the viability can be measured without stopping [92]. An important aspect to be considered in these and similar viability tests is the influence of the respective cell source (cell lines or primary cells) that may affect the results due to differences in metabolic activity for various cell types [90]. The MTT assay is often used for biocompatibility testing, while the AlamarBlue assay is commonly used for proliferation studies [93]. In this context, it was more relevant to use the latter for evaluating the antitumour effect on BCC cells (multiple time points in the same exposure experiment) and the MTT assay for evaluating the biocompatibility of the starting material (single time point according to commonly applied biocompatibility evaluations [26,94,95]). The test was executed for the drug-free polymer samples only (3PHEMA/PHPMA/NaDOC and 3PHEMA/PHPMA/NaDOC/FePt) (Figure 9a); the reason lies in the fact that 5-FU would also pose a cytotoxic effect against skin fibroblasts. This is also among the reasons to use localised drug delivery systems that effectively deliver the anticancer drug to the tumour site only [89,96,97].

The reason behind performing this test was mainly to determine the possible influence of the potentially formed harmful degradation byproducts that could contribute to impaired cell proliferation (i.e., lower cell viability) or by any other means affect the desired therapeutic effects of the prepared multilayer films. It was immediately evident that neither of the samples drastically altered the viability of the skin fibroblasts. However, the introduced FePt NPs had a slightly undesirable effect compared to the bare polymer multilayer film (*p*-values of < 0.05 for the base and the 1:2 dilution, respectively), which became negligible with dilution and, consequently, with a lower concentration of FePt NPs. More importantly, neither cytotoxic effects nor morphological changes (Figure 9b) were evident during the test. These results further prove the suitability of the developed multilayer nanofilms for targeted use.

Based on the results from functional testing, the fundamental findings that can be made here are listed below: (1) both 5-FU samples prepared exhibit anticancer properties against BCC cells, (2) a multilayered structure of 5-FU (nano)films allows for controlled release, and (3) no toxic degradation products were formed in any of the drug-free polymer matrices (all values are significantly above 50%), so the proposed system may provide a groundwork for further studies to develop a relatively safe approach to skin cancer therapy.

## 4. Conclusions

We have developed a repeatable and reliable multilayer nanofilm preparation process with a high potential for therapeutic interventions based on SMPNs and active pharmacotherapy with anticancer agents. The prepared composition demonstrated the ability of controlled drug release to avoid unwanted side effects, which is a superior feature of modern drug delivery systems. Furthermore, the multilayer structure enables simple yet effective dose variations, showing promise as a potential personalised treatment approach. Finally, we proved the beneficial therapeutic effect against basal-cell carcinoma and the safety of base matrices on human-derived skin fibroblasts. Overall, the developed multilayered nanofilms are promising candidates for skin cancer therapy, reducing severe unwanted side effects and improving therapeutic activity.

## Figures and Tables

**Table 1:** Overview of different multilayer thin film formulations.

#	Component	Mass in 1 mL of the Final Solution (mg)		
		PHEMA/PHPMA/NaDOC	PHEMA/PHPMA/NaDOC_5-FU	PHEMA/PHPMA/NaDOC/FePt_5-FU
1	PHEMA	3.617 mg	3.617 mg	3.617 mg
2	PHPMA	0.637 mg	0.637 mg	0.637 mg
3	NaDOC	0.013 mg	0.013 mg	0.013 mg
4	FePt	/	/	0.286 mg
5	5-FU	/	6.000 mg *	6.000 mg *

* The shown masses were applied to 1 mL of the 5-FU solution, which was applied as a separate layer to the sample, while the other components were part of the same solution/suspension.

**Table 2:** Optimal conditions and parameter settings of layer-by-layer (LbL) coating.

#	Parameters	CYCLE 1	CYCLE 2
1	Velocity (RPM)	1500	2500
2	Acceleration (RPM/s)	1000	1000
3	Duration (s)	50	30
4	Volume of drop (µL)	50	

**Table 3:** Contact angle measurement for our samples.

Sample	Average CA Value (°)	SD (°)
3PHEMA/PHPMA/NaDOC_5-FU_1	28.14	0.47
3PHEMA/PHPMA/NaDOC_5-FU_2		
3PHEMA/PHPMA/NaDOC/FePt_5-FU_1	24.78	0.91
3PHEMA/PHPMA/NaDOC/FePt_5-FU_2		
3PHEMA/PHPMA/NaDOC/FePt_1	21.89	1.18
3PHEMA/PHPMA/NaDOC/FePt_2		
3PHEMA/PHPMA/NaDOC_1	25.53	1.51
3PHEMA/PHPMA/NaDOC_2		

CA: contact angle; SD: standard deviation.

## Data Availability

All related data is part of the manuscript.
